# Illustrations of Cutaneous Diseases

**Published:** 1839-10

**Authors:** 


Art. XIII.—*Illustrations of Cutaneous Diseases.* By Robert Willis, m.d. Fasciculi IV, V, VI, VII, VIII.—*London,* 1839. Folio.

In our last Number we gave a brief notice of the first fasciculi of this work, and spoke somewhat disparagingly of them, as artistical productions. We condemned, also, in some respects, the meager plan adopted by the author, as scarcely allowing him to do justice either to his subject or to his own knowledge. We were fully aware that Dr. Willis's experience and great ability well qualified him for the task he had undertaken, and regretted that he had allowed himself to be hampered by a faulty plan, or a too thrifty publisher. The general defects of the plan still exist; but we are happy to observe that the character of the drawings, and still more of the colouring (so essential a point in all pathological illustrations) is considerably improved in the fasciculi now before us, more particularly in these last published. We observe, also, in Dr. Willis's brief annotations, so many indications of a good practical knowledge of his subject, that we have more cause than ever to regret the limitation imposed upon him by the original plan. We once more recommend this work to all our readers who feel the want of a guide through the obscure labyrinth of cutaneous diseases.

